# Rationale and Design of a Randomized Controlled Trial to Evaluate the Effectiveness of Medical Student Counseling for Hospitalized Patients Addicted to Tobacco (the MS-CHAT Trial)

**DOI:** 10.1155/2021/6682408

**Published:** 2021-01-27

**Authors:** Priyanka Satish, Aditya Khetan, Dharav Shah, Subhashini Ganesan, Rojith Balakrishnan, Shuba Srinivasan, Reema Samuel, Leland Hull, Richard A. Josephson

**Affiliations:** ^1^Department of Medicine, University Hospitals Cleveland Medical Center, Cleveland, OH, USA; ^2^Population Health Research Institute, McMaster University, Hamilton, ON, Canada; ^3^Alcohol and Drug Information Centre, Trivandrum, Kerala, India; ^4^Department of Community Medicine, PSG Institute of Medical Sciences and Research, Coimbatore, India; ^5^Department of Medicine, Government Calicut Medical College, Calicut, Kerala, India; ^6^Department of General Medicine, DM WIMS Medical College, Wayanad Dist., Kerala, India; ^7^Department of Psychiatry, Christian Medical College, Vellore, India; ^8^Division of General Internal Medicine, Massachusetts General Hospital, Boston, MA, USA; ^9^Harvard Medical School, Boston, MA, USA; ^10^Department of Cardiovascular Medicine, Case Western Reserve University School of Medicine, Cleveland, OH, USA

## Abstract

Globally, India is the second largest consumer of tobacco. However, Indian medical students do not receive adequate training in smoking cessation counseling. Each patient hospitalization is an opportunity to counsel smokers. Medical Student Counseling for Hospitalized patients Addicted to Tobacco (MS-CHAT) is a 2-arm multicenter randomized controlled trial (RCT) that compares the effectiveness of a medical student-guided smoking cessation program initiated in inpatients and continued for two months after discharge versus standard hospital practice. Current smokers admitted to the hospital are randomized to receive either usual care or the intervention. The intervention group receives inpatient counseling and longitudinal postdischarge telephone follow-up by medical students. The control group receives counseling at the discretion of the treating physician. The primary outcome is biochemically verified 7-day point prevalence of smoking cessation at 6 months after enrollment. Changes in medical student knowledge and attitude will also be studied using a pre- and postquestionnaire delivered prior to and 12 months after training. This trial tests a unique model that seeks to provide hands-on experience in smoking cessation counseling to medical students while simultaneously improving cessation outcomes among hospitalized smokers in India.

## 1. Introduction

Global Adult Tobacco Survey (GATS) 2016 showed that India had 100 million smokers [1]. Smoking cessation substantially reduces the risk of death associated with smoking, and interventions to quit smoking are both efficacious and cost-effective [2, 3]. Studies have shown that counseling hospitalized patients is effective if started during hospitalization and continued for at least a month after discharge [4]. However, numerous barriers exist to widespread adoption of this practice [5].

Most physicians lack the necessary knowledge and skills to offer effective cessation counseling to their patients [6, 7]. This gap likely starts from medical school [8]. Purely didactic-based teaching may not be sufficient in preparing students for independent practice, particularly if inadequately supplemented with the varied experience of counseling actual patients [9]. Educational interventions have been found to have greater student engagement when they include a component of integrative learning, such as in a real patient encounter [10].

We designed the current study to test the hypothesis that utilizing trained medical students to counsel hospitalized smokers will lead to an increase in patient quit rates, while also improving medical student knowledge and confidence regarding smoking cessation counseling.

## 2. Materials and Methods

The Medical Student Counseling for Hospitalized patients Addicted to Tobacco (MS-CHAT) study protocol received institutional approval from the institutional review board at University Hospitals/Case Western Reserve University, USA, as well as the sites in India. The trial was registered in the https://clinicaltrials.gov/ database on 10 May 2018 (NCT03521466). The trial started recruiting in December 2018. It is expected to continue until July 2021. All patients enrolled in the trial will provide written informed consent. The operational definitions for the terms used in the methods and outcome sections are given in Table 1.

### 2.1. Medical Student Education

#### 2.1.1. Participants

**Figure 1:**
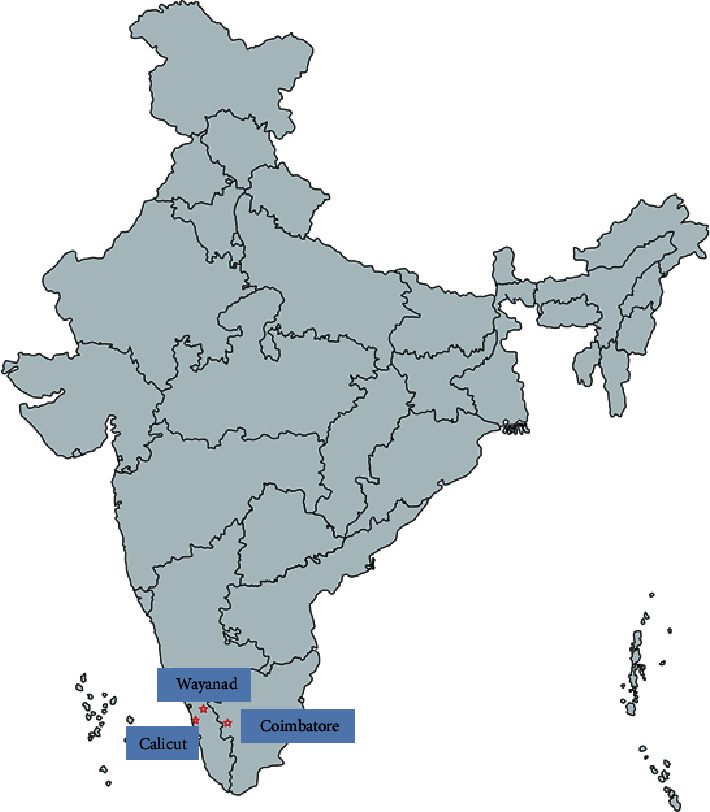
Location of study sites in India.

Second-year students from three different medical schools (Figure 1) are called upon to volunteer for the program, with 30 students from each school enrolled in the study. These medical schools train between 150 and 250 students in each class. The medical school course in the participating medical schools is of 5.5-year duration. Medical and surgical clinical rotations start during the students' second year with case-based discussion, tailored to the level of training.

#### 2.1.2. Training

The training modules were developed by adapting the WHO guide for tobacco cessation counselors to the Indian context. The content predominantly addresses the skills of behavioral counseling. The training consists of a three-hour didactic lecture, followed by group role-playing scenarios for two hours, with peer and proctor feedback. Following training, the students are asked to complete a knowledge questionnaire. Students are selected for the second part of the trial (patient counseling phase) once they obtain a minimum test score.

### 2.2. Patient Counseling

**Figure 2:**
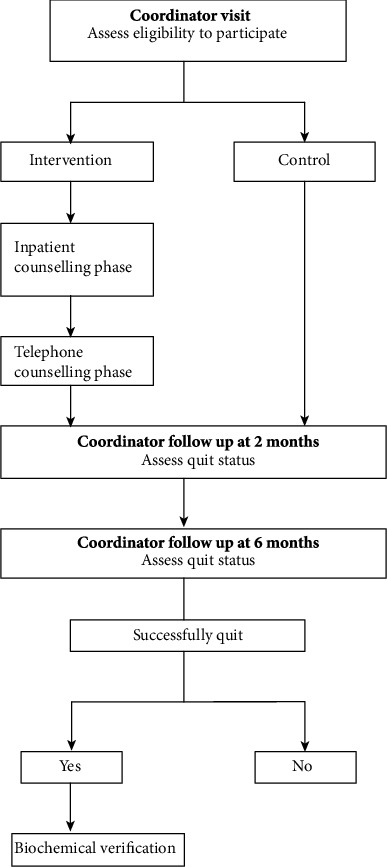
Structure of study.

The structure of the study is described in Figure 2.

#### 2.2.1. Trial Design and Participants

This is an open-label, two-armed, parallel-group, block randomized controlled trial with 1 : 1 concealed allocation. Patients are the unit of randomization. Eligibility criteria include age 18-70 years, active admission to the hospital, and current smoking or report having smoked in the last four weeks prior to admission (to account for changes in behavior during illness). Eligible patients are stratified based on the medical school and block randomized into an intervention or control group using a block size of 20. Opaque envelopes developed by the research team are provided to the coordinators to be used for randomization.

Exclusion criteria include patients using only nonsmoked tobacco and those who are daily alcohol users or daily drug users. Patients deemed unable to follow-up, either because of distance from the hospital or because of psychiatric, social, or medical factors, will be excluded. Patients currently participating in another tobacco cessation program are also excluded.

#### 2.2.2. Intervention Group

Participating medical students offer inpatient counseling to patients in the intervention group once during their hospitalization (recommended duration of 15-20 minutes). The medical students then follow-up with their patients and provide telephone counseling (a minimum of three sessions of 15 minutes each, over two months).

#### 2.2.3. Control Group

Smoking cessation advice is left to the discretion of the treating physician, to reflect usual care.

### 2.3. Data Collection

A study coordinator, who is blinded to group assignment, will collect follow-up information from patients in both the intervention and control groups at six months after enrollment. Patients who claim to have stopped smoking (not a single puff in the last seven days) at six months will be called in for an exhaled breath carbon monoxide (CO) test. The follow-up procedure will be identical in both groups.

Focus group discussions will be conducted among patients at the end of the trial to analyze patient knowledge, attitudes, and behaviors towards smoking cessation. Focus group discussions of students will also be conducted to qualitatively understand their experience of the program.

### 2.4. Study Outcomes

The primary outcome measure will be a biochemically verified seven-day quit rate at six months from enrollment. The criteria for a verified quit attempt will be an exhaled CO level of <10 PPM. The participant is blinded to the definition of the primary outcome (biochemically verified seven-day quit rate) until the follow-up visit is underway. Planned secondary and descriptive outcomes are listed in Tables 2 and 3, respectively.

### 2.5. Sample Size and Statistical Analysis

We calculated the sample size needed for a study with a power of 85%, *α* of .05 to detect a 10% absolute difference in the primary outcome assuming a control quit rate of 20%. This was based on a prior meta-analysis of tobacco cessation interventions among hospitalized smokers [4]. We assumed an attrition rate of 20%. Based on these calculations, a sample size of 830 patients will be required, resulting in 415 patients in the intervention and a similar number of patients in the control group.

The primary outcome will be analyzed by an intention-to-treat approach. Patients lost to follow-up at any time point will be considered as smokers for analysis. A secondary analysis with smoking status at last phone contact will be performed.

## 3. Results and Discussion

The MS-CHAT trial will test if trained medical students can improve smoking cessation outcomes among hospitalized patients who smoke tobacco. This is a potentially scalable, low-cost model that combines medical student skill development with inpatient smoking cessation counseling. Providing experiential training to medical students has the potential to increase the delivery of smoking cessation counseling when they step into practice, by increasing their comfort with this skill. Furthermore, the skills acquired in MS-CHAT may enable medical students to provide counseling for diet, physical activity, and other behavioral barriers to optimal health [11].

## 4. Conclusions

The findings of this trial will have implications for noncommunicable disease intervention strategies not just in other low- and middle-income countries but potentially also in advanced health care systems tackling some of the same issues.

## Figures and Tables

**Table 1:** Definitions.

7-day quit rate: defined as the patient's self-reported smoking behavior in the last 7 days. Assessed as “Have you refrained from smoking during the past seven days, and not smoked even one puff?”
Continuous abstinence: defined as being abstinent from smoking for at least the prior 90 days. Patients will be asked to specify quit date or number of weeks/months since quitting.
Biochemical testing: breath carbon monoxide < 10 PPM constitutes a verified successful quit attempt.
Previous quit attempt: a 24-hour quit attempt in the past 12 months.

**Table 2:** Planned secondary outcomes.

Patient outcomes:
(i) Biochemically verified 90-day quit rate, measured 6 months after enrollment. (ii) Number of patients achieving a 50% reduction in the number of cigarettes/bidis smoked in a week, measured at 2 and 6 months after enrollment. (iii) Correlation between verified quit rates at 6 months and FTND scores at enrollment. (iv) Correlation between verified quit rates at 6 months and number of quit attempts, by the study group. (v) Number of patients who report having quit smoking in the last 7 days at 2 and 6 months from enrollment (irrespective of biochemical verification). (vi) Agreement between self-reported 7-day quit rate and biochemically verified 7-day quit rate, measured at 6 months from enrollment. (vii) No. of patients who have refrained from using smokeless tobacco in the last 7 days, measured at 6 months from enrollment. (viii) Per protocol analysis of the 7-day quit outcome, 6 months after enrollment. Patients in the intervention arm who did not receive a hospital counseling session and at least two follow-up phone calls will be excluded.
Medical student outcomes:
(i) Pre-post analysis of medical student knowledge about counseling techniques and pharmacotherapy before, 6 weeks, and 12 months after training. (ii) Correlation between scores on the knowledge survey with patient quit rates. (iii) Qualitative analysis of the effectiveness of the training modules assessed by student survey, administered 6 weeks after training. (iv) Qualitative analysis of student attitudes towards smoking cessation counseling as assessed by a survey, administered 12 months after training. (v) Qualitative analysis of the trial design and implementation as assessed by a survey administered 12 months after training.

**Table 3:** Planned descriptive outcomes.

Descriptive outcomes: (i) Number of enrolled patients that receive inpatient counseling and at least 3 follow-up calls, calculated at 2 months from enrollment. (ii) Number of patients who use any form of cessation pharmacotherapy within the first 2 months of enrollment. (iii) Number of patients enrolled in the study that are available for follow-up at 6 months from enrollment.

## Data Availability

There is no data collection associated with this paper.
